# Draft genome of methanol-oxidizing *Methylobacterium fujisawaense* strain LAC1

**DOI:** 10.1128/MRA.00331-23

**Published:** 2023-09-20

**Authors:** Jooho Chung, Jinha Kim, John C. Blazier, Myung Hwangbo, Kung-Hui Chu

**Affiliations:** 1 Zachry Department of Civil and Environmental Engineering, Texas A&M University, College Station, Texas, USA; 2 Texas A&M Institute for Genome Sciences and Society, Texas A&M University, College Station, Texas, USA; 3 School of Earth, Environmental and Marine Sciences, The University of Texas - Rio Grande Valley, Brownsville, Texas, USA; University of Southern California, Los Angeles, California, USA

**Keywords:** *Methylobacterium fujisawaense*, rare earth elements, methanol oxidation, acidophiles

## Abstract

We report the draft genome of *Methylobacterium fujisawaense* LAC1 isolated from an acidic aquifer in Indian Head, MD, USA. The genome contains 5,883,000 bp and has a GC content of 70% with 5,434 protein-encoding genes with functional assignments. This strain can grow on methanol with lanthanum, a rare earth element.

## ANNOUNCEMENT

*Methylobacterium fujisawaense* LAC1 (designated as LAC1 hereafter) is an acidophilic, pink-pigmented methylotrophic bacterium that can grow on methanol in acidic medium (pH 5.5) with lanthanum (La), a rare earth element. LAC1 was isolated from an enrichment culture inoculated with the groundwater of a contaminated acidic aquifer site at the Indian Head Division Naval Surface Warfare Center, Charles County, MD. The enrichment culture was established in calcium- and copper-free ATCC 2157 medium (1) with 0.1% methanol and 30 µM La^3+^ at room temperature. ATCC 2157 medium was prepared without the addition of calcium and copper salts. The enrichment culture was then incubated with shaking at room temperature. Following several respikes of methanol, a loopful of the enrichment culture was used to streak on Gelrite (RPI, IL) plates containing the same medium with 0.5% methanol and 30 µM La^3+^. After restreaking several times, a pure colony of LAC1 was obtained. The genomic DNA of LAC1 was extracted using a FastDNA SPIN kit for soil (MP biomedical, CA), and the extracted gDNA quantity was determined using the Qubit High Sensitivity (HS) Assay Kit on the Qubit Fluorometer 4.0 (Invitrogen, CA), and the integrity was confirmed by the Genomic DNA ScreenTape Assay Kit (Agilent, CA) on the 4200 TapeStation (Agilent, CA). Library of LAC1 was prepared using Illumina DNA Prep Kit with Illumina IDT for Illumina UD indexes (Plate A; Illumina, CA). The same HS Assay Kit was used for post-library quantification and quality controlled by D1000 ScreenTape Assay Kit (Agilent, CA) on the 4200 TapeStation. Sequencing was conducted by the Illumina MiSeq platform (Illumina, CA) with the paired-end 2 × 250 bp strategy, resulting in a total of 518,277 paired-end reads. The reads were trimmed, adapter sequences were removed, and quality control was performed using FastQC within Trim Galore v0.6.7 (2). Sequence reads were assembled *de novo* in SPAdes v3.15.3 (3) using the “isolate” setting, on the Texas A&M University’s Grace computing cluster. BLAST search and BV-BRC’s MinHash k-mer-based “Similar Genome Finder” tool were used to identify the isolate as the most closely related to *Methylobacterium radiotolerans* strain JCM 2831. Annotation of the assembly with the RASTtk-based (4) custom annotation pipeline at BV-BRC (5) indicated a high level of contamination (>10%). The SPAdes contigs showed a bimodal distribution of k-mer coverage values, with one group of contigs having k-mer coverage values ~ 8 and another group with k-mer coverage values ~2. Removal of the lower k-mer coverage contigs using a publicly available Bash one liner (https://github.com/ECBSU/oneliners) yielded a high-quality (high completeness and low contamination) assembly when annotated again with the custom annotation pipeline at BV-BRC. The filtered contigs were then submitted and annotated through NCBI PGAP v4.6 (6). Genome coverage was calculated by SAMtools v1.16.1 (7) after mapping the reads to the assembled genome using BWA-MEM2 v2.2.1 (8). Default parameters were used for all software unless otherwise specified.

The assembled draft genome of LAC1 is 5,883,000 bp long with 28.0× genome coverage and 70% GC content. It consists of 72 contigs, with an N_50_ value of 185,086 bp. The genome contains 5,556 total genes including 5,434 total protein-encoding genes, 3 5S rRNA genes, 1 16S rRNA gene, 1 23S rRNA gene, and 49 tRNA genes. The 16S rRNA gene sequence of LAC1 has 92% similarity to *M. fujisawaense* DSM 5686.

## Data Availability

The draft genome sequence was deposited in GenBank under the accession number JARWLY000000000. The BioProject, BioSample, and SRA accession numbers are PRJNA948434, SAMN33903885, and SRR24019894, respectively.
